# Excisional Wound Healing Is Delayed in a Murine Model of Chronic Kidney Disease

**DOI:** 10.1371/journal.pone.0059979

**Published:** 2013-03-25

**Authors:** Akhil K. Seth, Mauricio De la Garza, Robert C. Fang, Seok J. Hong, Robert D. Galiano

**Affiliations:** Laboratory for Wound Repair and Regenerative Medicine, Division of Plastic and Reconstructive Surgery, Feinberg School of Medicine, Northwestern University, Chicago, Illinois, United States of America; University of Louisville, United States of America

## Abstract

**Background:**

Approximately 15% of the United States population suffers from chronic kidney disease (CKD), often demonstrating an associated impairment in wound healing. This study outlines the development of a surgical murine model of CKD in order to investigate the mechanisms underlying this impairment.

**Methods:**

CKD was induced in mice by partial cauterization of one kidney cortex and contralateral nephrectomy, modifying a previously published technique. After a minimum of 6-weeks, splinted, dorsal excisional wounds were created to permit assessment of wound healing parameters. Wounds were harvested on postoperative days (POD) 0, 3, 7, and 14 for histological, immunofluorescent, and quantitative PCR (qPCR).

**Results:**

CKD mice exhibited deranged blood chemistry and hematology profiles, including profound uremia and anemia. Significant decreases in re-epithelialization and granulation tissue deposition rates were found in uremic mice wounds relative to controls. On immunofluorescent analysis, uremic mice demonstrated significant reductions in cellular proliferation (BrdU) and angiogenesis (CD31), with a concurrent increase in inflammation (CD45) as compared to controls. CKD mice also displayed differential expression of wound healing-related genes (VEGF, IL-1β, eNOS, iNOS) on qPCR.

**Conclusions:**

These findings represent the first reported investigation of cutaneous healing in a CKD animal model. Ongoing studies of this significantly delayed wound healing phenotype include the establishment of renal failure model in diabetic strains to study the combined effects of CKD and diabetes.

## Introduction

The impact of chronic kidney disease (CKD) on health care costs has received increased attention over recent years [1]–[7]. In fact of 1.2% of Medicare patients in the United States were found to have CKD in 2005, which represented a disproportionate share of total Medicare costs at 6.4% [7]. According to the most recent United States Renal Data System Annual Report, approximately 14–16% of the U.S. population suffers from CKD (United States Renal Data System, 2011 Atlas of CKD, http://www.usrds.org/atlas.aspx). Unfortunately, this number continues to rise, due in part to widened access to dialysis, along with a concurrent increase in the prevalence of hypertension and diabetes [6].

As part of its systemic impact, CKD leads to pleiotropic changes in the skin, including dryness, rashes, microangiopathy, and even calciphylaxis, for which a direct correlation exists with the severity and duration of the CKD state [8], [9]. This is further complicated by the association between CKD and other pervasive, chronic co-morbidities that impact wound healing, such as peripheral vascular disease and diabetes [10]–[12]. Given this complexity, impaired wound healing in this population represents a challenge to clinicians, with difficult to treat pathologies such as chronic open wounds, venous ulcers, and critical limb ischemia [13]–[15]. Consequently, patients' prognoses are often poor, with many suffering significant morbidity including extremity amputation [16].

With CKD and wound healing both being multi-cellular and multi-organ processes that involve the vascular system, immune system, skin, and growth factors, there is no ideal way to study their intricate interaction outside of an animal model. Therefore, the refinement of animal models has remained an essential component to the research surrounding both of these processes [17]–[26]. To date, a variety of models have been developed to simulate human CKD pathophysiology. Non-surgical approaches include the administration of pharmacological substances, such as uranium nitrate in dogs [17] or cisplatin in rats [18]. In contrast, surgically-based methods of inducing renal failure include injuring the kidneys, resecting a kidney, or a combination of both techniques [19]–[23]. Similarly, *in vivo* models of wound healing have been integral to advancing our understanding of normal wound healing processes and the pathologies that impact their natural progression [24]–[26]. In particular, the senior author has previously refined an excisional, murine model of wound healing through the use of cutaneous splints, which allows for wound reepithelialization by minimizing the contraction typically seen in rodent wounds [25]. This allows for more clinically translatable wound healing research while taking advantage of the assortment of genetic and molecular tools available for murine-based research.

Despite an established knowledge base surrounding both CKD and wound healing, only a paucity of literature exists addressing the mechanistic link between CKD, and the phenotypic impairment of wound healing [17], [27]–[31]. In particular, current *in vivo* models of these two concurrent processes are lacking, with the majority developed more than 15–40 years ago without any further refinement [17], [28]–[31]. This lack of research progress stands in contrast to the growing burden of chronic wounds and CKD on patient quality of life [32]–[34]. In an effort to address this growing need for continued research, we have developed a robust and consistent murine model of chronic wound healing in a CKD background. By modifying a previously described method for renal failure induction by Gagnon et al [22], within our splinted, cutaneous wound healing model [25], we aimed to understand the extent to which CKD-based uremia impairs normal wound healing. Furthermore, we performed initial studies into the mechanisms underlying these impairments, providing a foundation for future therapeutic development and testing.

## Methods

### Animals

CKD-inducing surgical procedures were performed in 8–10 weeks old male C57BL/6 inbred mice obtained from Jackson Laboratories (Bar Harbor, ME, USA). The animals were acclimatized to their environment for at least 1-week before the initial procedure. Throughout the totality of the experiment all animals were fed with the same standard mouse pellet diet, received water ad libitum, and were maintained in a temperature-controlled animal facility with a 12-hour light/dark cycle. All mice were administered pre-operative analgesia 30-minutes prior to the surgical procedures via a subcutaneous injection of buprenorphine (0.5 mg/kg). Anesthesia was achieved by means of 1.5–2.5% inhaled isofluorane at a flow rate of 1–2 liters per minute. Experimental groups for both the chronic kidney disease and control mice consisted of n = 6–8 mice for each time point. CKD mice were defined as C57BL/6 mice that underwent CKD-induction surgery, while control mice were defined as equivalently old C57BL/6 mice with uninjured kidneys.

**Figure 1:**
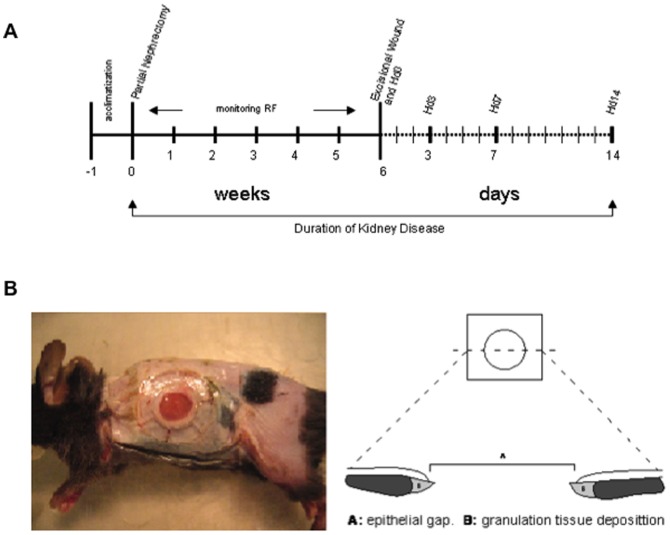
Outline of experimental protocol with dates of procedures indicated and splinted excisional wound model. A) RF = Renal Failure, Hd = Harvest day. B) The splinted excisional wound model minimizes the role of wound contraction during rodent wound healing, allowing for a finer analysis of parameters such as epithelial gap closure and granulation tissue deposition.

Each mouse had two dorsal splinted excisional wounds, as previously described [25], allowing for a total of 12–16 wounds for analysis per time point. Their ability to thrive throughout the entire experiment was assessed by survival, growth rate and general subjective well-being. Mice were housed up to 5-animals per cage before and after the partial nephrectomy. CKD mice were continuously monitored during progression into their CKD state for at least 6-weeks before the initiation of wound healing studies. Mice were then caged alone after the splinted excisional wounds were created on the dorsum to permit quantification of wound healing parameters each individual mouse. Wounds were harvested on days 0, 3, 7, and 14 for histologic, immunofluorescent, RNA, and protein analyse (Figure 1A). All animal experimentation described in the manuscript was conducted in accordance with the National Institutes of Health Guide for the Care and Use of Laboratory Animals using protocols approved by the Institutional Animal Care and Use Committee of Northwestern University School of Medicine. The animals utilized in this experiment all received humane care.

### Induction of Chronic Kidney Disease

CKD was induced using a modification of the partial nephrectomy technique previously described by Gagnon et al [22]. Our primary modification was to perform both the nephrectomy and contralateral injury concurrently during one procedure. We attribute the feasibility of the surgical model mainly to the use of inhaled anesthesia, which decreases the physiologic burden on the animal as compared to intraperitoneal anesthesia. The result is a reduction in peri-operative morbidity to almost null. Briefly, through a small flank incision, the left renal hilum was surgically exposed and tied with a 5-0 Vicryl suture proximal to the kidney. The kidney was then removed while maintaining hemostasis. Immediately following removal, the right kidney was exposed in a similar fashion. However, following clearance of the renal capsule, the renal pedicle was briefly clamped with subsequent electrical cauterization of 50–75% of the renal cortex so that at least 1-mm around the hilum was left free of injury. During both procedures, special care was taken not to manipulate the ureters. Incisions were closed using a standard two-layer approach, including closure of the peritoneum and body wall muscle with a 6-0 Vicryl suture and then skin approximation with 6-0 nylon sutures. All mice received a 1-ml bolus of intraperitoneal phosphate-buffered saline (PBS) immediately postoperatively. After completing the procedure, the animals were placed on a warming pad and monitored until full recovery for 5–10 minutes. The total duration of the surgery never exceeded 20 minutes. Renal function was routinely monitored until sacrifice day through analysis tail vein blood for quantitative colorimetric urea using a Quantichrom Urea Assay (DIUR-500, Bioassay Systems, Hayward, CA, USA) and a μQuant microplate spectrophotometer (Bio-tek Instruments, Winooski, VT, USA). Body weight was recorded routinely using a conventional Triple Beam Balance scale (OHAUS, Florham Park, NJ, USA). Hemoglobin levels were also measured intermittently using a Hemoglobin Colorimetric Assay kit (Quantichrom from Bioassay Systems, Hayward, CA, USA).

### Experimental Wound Model

After a minimum of 6-weeks post-partial nephrectomy, mice underwent splinted excisional wound creation while being maintained in a chronic renal failure state. As previously described by Galiano et al [25], this model has the advantage of minimizing rodent wound contraction, ensuring that healing occurs by granulation tissue deposition and epithelialization. The resultant wounds allow for improved interrogation of the processes important in human chronic wound healing (Figure 1B). Briefly, a sterile 6-mm biopsy punch was dipped in marking ink and pressed against the shaved skin in the dorsum to mark the line of incision. A sharp iris scissors was used to cut out a 6-mm circular wound from the skin, including the panniculus carnosus layer. A second wound was made in a similar fashion on the opposite side of the dorsal midline. An 8-mm in inner diameter circular silicone splint was fixed to the skin using a silicone immediate-bonding adhesive (NO TAPE, Vapon Inc, Fairfield, NJ, USA) and then sutured onto the skin just beyond the wound periphery using interrupted 6-0 nylon sutures in a horizontal mattress fashion. The wounds were then covered with a small piece of semi-occlusive dressing (TegaDerm, 3 M, St. Paul, MN, USA). A thin protective dressing (Wet-Pruf, Kendall, Mansfield, MA, USA) was then placed around the surgical site to prevent the mouse from marring either the semi-occlusive dressing or the silicone ring with their normal grooming behaviors. Procedure length is approximately 20 minutes, followed by a 5–10 minute anesthetic recovery time. Animals were then caged separately until they reached their respective time point for wound analysis.

### Kidney Glomerular Tuft Area

After at least 6-weeks of being in a CKD state, 6–8 mice were sacrificed for kidney glomerular tuft area analysis. For pathohistological evaluation, kidneys were cut longitudinally at the time of animal sacrifice and fixed with 10% buffered formalin phosphate (Fisher Scientific, Pittsburg, PA, USA) overnight. Sections were embedded in paraffin, cut into section 6-microns in thickness, and stained with hematoxylin and eosin (H&E). Glomerular images were digitalized using a color video camera attached to a microscope (NIS-Elements BR 2.30, Nikon Instruments Inc., Melville, NY) at 400× magnification. After capture, the glomerular tuft was traced digitally, and the areas were calculated using computer image analysis software (NIS-Elements BR 2.30, Nikon Instruments Inc., Melville, NY). Indirect signs of mesangial expansion were assessed by glomerular hypertrophy, quantified by measuring the glomerular tuft cross-sectional area from glomeruli in which the vascular pole was evident (using at least 20 glomeruli per section). This was performed to reduce the probability of including tangentially cut glomeruli.

### Histology and Wound Analysis

Wounds were harvested on days 0, 3, 7 and 14, for histological analysis. At the time of sacrifice, a full thickness circular excision was made around each wound, including a surrounding 1 mm of healthy non-wounded normal skin. Wounds were bisected, with fixation of one semicircular wound section in 10% buffered formalin phosphate (Fisher Scientific, Pittsburg, PA, USA) for 6 hours, followed by embedding in paraffin. The remaining half of the wound was used for immunofluorescence analysis, as described below. Paraffin embedded tissues were cut using a Microm HM 315 histological microtome (Thermo Fisher Scientific Inc., Kalamazoo, MI) at 6-microns in thickness, followed by placement on positively charged Superfrost-Plus micro slides (VWR International, West Chester, PA). Histological wound healing parameters including epithelial gap closure and granulation tissue deposition area were measured after staining with H&E from digital images at 20× magnification with an Eclipse Phase 50i upright digital microscope (Nikon Instruments Inc., Melville, NY). The percentage of epithelial gap closure and granulation tissue area were quantified using NIS-Elements BR 2.30 computer image analysis system (Nikon Instruments Inc., Melville, NY). Epithelial gap was defined as the distance between the advancing edges of keratinocyte migration across the wound and calculated as a percentage of the total epithelial closure of the wound. A wound was considered completely reepithelialized when the epithelial gap was equal to zero. Because the wound edges are kept at a constant length with the splint, these were used to normalize the epithelial closure. Area of granulation tissue was calculated by digitally outlining the regions of granulation tissue and then calculating pixel area. The total area of granulation tissue was the sum of these regions.

### Immunofluorescence

In addition to H&E processing, 6–8 wounds per group were harvested on days 0, 3, 7 and 14, for immunofluorescence analysis. Following the aforementioned excision protocol, half of the wound was immediately embedded with OCT compound (Sakura Finetek USA Inc., Torrance, CA) in a 15 mm×15 mm×5 mm plastic cryomold (Sakura Finetek USA Inc., Torrance, CA) and frozen with liquid nitrogen. Frozen tissue cross-sections were then cut to 5-microns in thickness from the mid-portion of each wound using a CM1850 cryostat (Leica Microsystems Inc., Bannockburn, IL), and placed on positively charged glass slides. The frozen sectioned tissue samples were then individually stained with a M20107S sheep anti-BrdU primary antibody (Meridian Life Science Inc., Saco, ME) at a 1∶125 dilution to quantify cell proliferation, PECAM-1 553370 rat anti-mouse CD31 primary antibody (BD Biosciences, San Jose, CA) at a 1∶50 dilution to assess vessel density, and with 550539 rat anti-mouse CD45 primary antibody (BD Biosciences, San Jose, CA) at a 1∶50 dilution to quantify inflammatory cells. All slides were co-stained with P-36931 anti-fade DNA selective nuclei DAPI staining compound (Invitrogen, Carlsbad, CA) for cell identification reference.

### Tissue Gnostics quantification and statistical analysis

Quantification of BrdU, CD31 and CD45 positive cells was performed using TissueFAXS (Tissue Gnostics, Los Angeles, CA), a computerized, high profile, multi-channel microscopic system that scans immunofluorescence stained whole sections and performs quantitative analysis of staining intensities, allowing for automated whole single cell detection. Microscopic thresholds were set so that each immunofluorescent marker-positive cell was identified only when co-stained with a DAPI-positive cell. Utilizing TissueQuest (Tissue Gnostics, Los Angeles, CA) analysis software, the total marker-positive individual cells were quantified in whole wounded tissues. Results were presented as mean value ± standard deviation, with statistical analyses performed using an unpaired, student's t-test for continuous variable comparisons. A *p* value of less than 0.05 was considered statistically significant.

### Quantitative Reverse-transcription PCR

Total RNA was also isolated from wound bed tissues for RNA extraction and quantitative reverse-transcription PCR (qRT-PCR) analysis. Wound samples were obtained from 4 CKD mice and 4 control mice per group. Samples were harvested on days 0, 3, and 7 following dorsal splinted wound creation. Wound samples were homogenized using a Mini-bead beater-8 equipment (Biospec Products Inc, Bartlesville, OK) using the Zirconia beads (2.0 mm diameter, Biospec Products Inc) in the presence of Trizol Reagent (Sigma-Aldrich, St. Louis, MO). Total RNA was isolated according to the manufacturer's protocol. Contaminating genomic DNA during RNA preparation was removed using the Turbo DNA-free kit (Ambion, Austin, TX). cDNA was generated from 5-µg of total RNA using superscript II (Invitrogen, Carlsbad, CA) with 100-ng of random primers (Invitrogen). For quantitative analysis of the expression level of mRNAs, real-time PCR analyses using SYBR green I were performed using an ABI prism 7000 sequence detection system (Applied Biosystems, Foster City, CA). PCR primers were designed using the Primer 3 program (http://frodo.wi.mit.edu/) or selected from the PrimerBank database (http://pga.mgh.harvard.edu/primerbank/). The amplifications were performed in 25-µl vials containing 0.2 µM each primer, 0.5 X SYBR Green I (Molecular Probes, Eugene, OR), and 1.5 µl of 5 fold diluted cDNA. Expression of each gene was normalized to the level of GAPDH (glyceraldehyde 3-phosphate dehydrogenase) to obtain a ΔCt. The 2-ΔΔCt method was used to calculate gene expression difference in the wounded skin of CKD and control mice, with subsequent days (days 3 and 7) being than expressed as the fold difference over day 0 of the control wound. Expression of genes was detected by PCR with the following oligonucleotides: GAPDH: 5′- TGACATCAAGAAGGTGGTGAAGC-3′ and 5′-CCCTGTTGCTGTAGCCGTATTC-3′, VEGF: 5′-GTACCTCCACCATGCCAAGT-3′ and 5′- ACACAGGACGGCTTGAAGAT-3′, PDGF-β: 5′-GATCTCTCGGAACCTCATCG-3′ and 5′-GGCTTCTTTCGCACAATCTC-3′, eNOS: 5′-GACCCTCACCGCTACAACAT-3′ and 5′-CTGGCCTTCTGCTCATTTTC-3′, iNOS: 5′-GTTCTCAGCCCAACAATACAAGA-3′ and 5′-GTGGACGGGTCGATGTCAC-3′, IL-1β: 5′-TGTGAAATGCCACCTTTTGA-3′ and 5′- TGTCCTCATCCTGGAAGGTC-3′, TNF-α: 5′-CGGACTCCGCAAAGTCTAAG-3′ and 5′-CGTCAGCCGATTTGCTATCT-3′.

## Results

### Single-stage renal injury and contralateral nephrectomy is a viable surgical model that produces consistent CKD in mice

**Figure 2:**
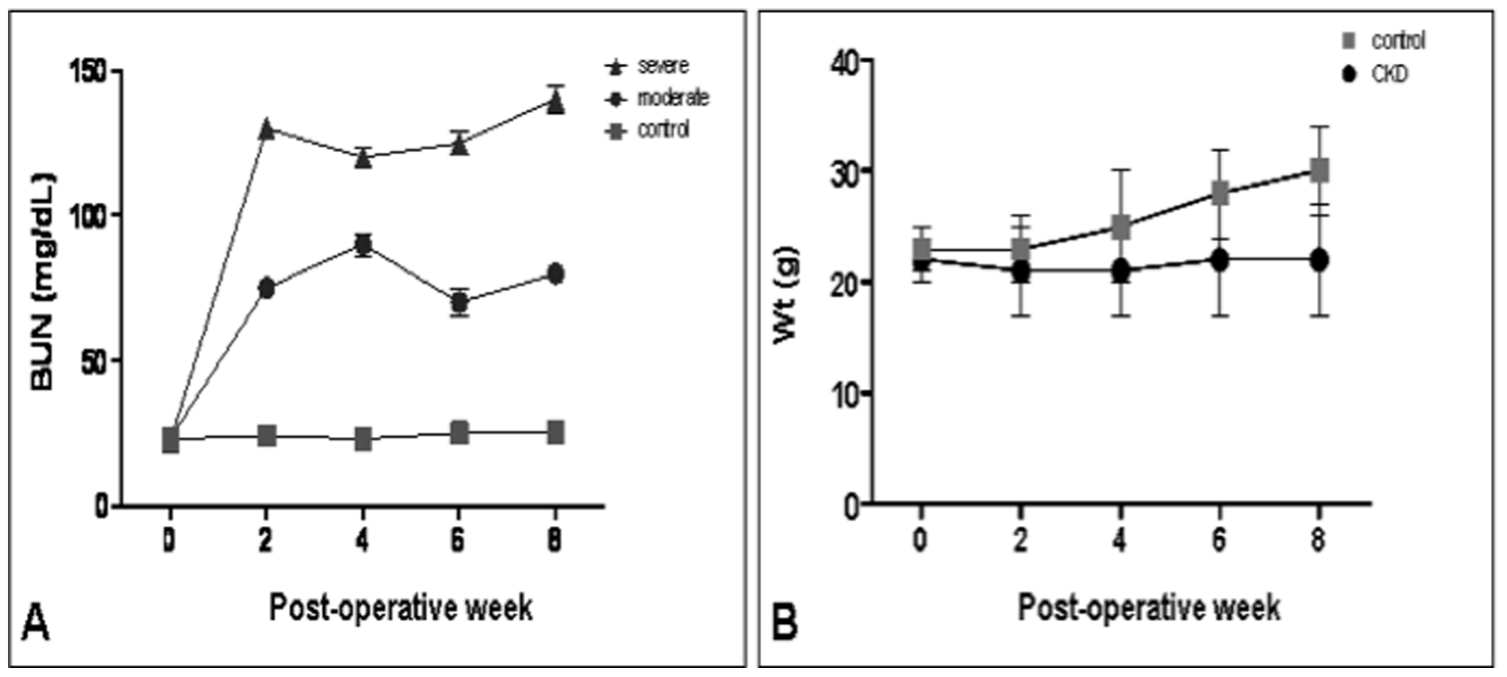
Mice exhibited different degrees of chronic kidney disease. **A**) By controlling the amount of cautery to the injured kidney, moderate and severe degrees of chronic kidney disease were produced. Only mice with a severe degree of chronic kidney disease were wounded for experimental purposes. (n = 6–8 mice/group) **B**) These animals exhibited metabolic derangements analogous to those in human chronic kidney disease and a notable growth retardation.

Our modification of the surgical technique originally described by Gagnon et al [22], which combined the stages of nephrectomy and contralateral renal injury, did not lead to an unacceptable mortality rate. Furthermore, we successfully created moderate and severe renal failure groups by controlling the degree of electrocautery applied to the preserved kidney. The moderate renal failure group had a mean BUN level of 80±20 mg/dl (52–104 mg/dl), while the severe renal failure group had a mean BUN level of 130±20 mg/dl (105–155 mg/dl) (Figure 2A). Our surgical technique was free of local complications, and we did not have any peri-operative deaths secondary to inhaled anesthesia.

Despite carefully limiting the degree of electrocautery for the severe CKD group, BUN levels above 155 mg/dl were found in 20% of the animals. These mice displayed protracted lethargy, poor hair grooming, and aversion to food only 2–3 days after the partial nephrectomy. Since death appeared imminent for these mice, they were properly euthanized and excluded from analysis. Death was ascribed to the inability of the remaining kidney to satisfy the animal's metabolic demands. Autopsy performed in all instances revealed no gross intra-peritoneal abnormalities, no signs of bleeding, intestinal obstruction or peritonitis. In all CKD mice, the flank wounds were completely healed and with no signs of dehiscence, inflammation, infection or erythema detectable. All 80% of the operated mice that survived the CKD producing surgery with inclusive severe chronic kidney disease BUN levels demonstrated normal behaviors, level of activity, and an overall appearance comparable to control mice throughout the remainder of the experimental protocol. The only exception to this was a reduction in hair growth rates at the site of surgical flank shaving.

The effects of CKD on growth and body weight were readily observed, with serial measurements shown in Figure 2B. CKD mice showed significant growth retardation throughout the study. Along with their profound uremia, these mice also exhibited deranged hematological profiles. Gagnon et al sequentially evaluated CKD mice from the 1^st^ to the 15^th^ week of renal failure, observing that anemia was established by the 2^nd^ week after surgery [35]. This CKD anemia is comparable to that of humans, in that it is normocytic, normochromic, with the mice possessing normal serum iron levels and stores. In order to corroborate the anemia in our model, we serially measured the hemoglobin concentration of both groups every 2-weeks. Mean hemoglobin concentration in control mice was 13.9±0.9 g/dl, while CKD mice showed a significant reduction in the concentration of hemoglobin to 10.4±1.5 g/dl at 2 weeks post CKD-inducing surgery. This level was maintained for the remainder of the experimental period (data not shown).

### CKD kidneys exhibit an increased glomerular tuft area

**Figure 3:**
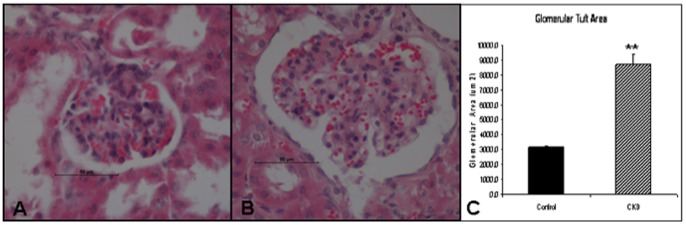
Chronic kidney disease (CKD) mice exhibited a significant increase in glomerular tuft area. Using hematoxylin and eosin to stain the paraffin embedded kidneys glomerular tuft area was measured from glomeruli in which the vascular pole was evident to compare the control kidneys (**A**) to the CKD kidneys (**B**). CKD kidneys exhibited indirect signs of mesangial expansion with a significant increase in glomerular tuft area when compared to control kidneys. (Magnification ×400) (** *p*<0.01) (n = 6–8 mice/group).

Gross examination of the peritoneal cavity of CKD mice revealed enlarged right kidneys surrounded by firm adhesions along the flanks, none of which appeared to obstruct intestinal flow or interfere with mouse intestinal physiology. No other abnormalities were noted outside of the kidneys. Post-mortem histological sections of kidney glomeruli stained with H&E after 6-weeks of CKD, and their representative control, are shown in Figures 3, A and B, respectively. Kidneys from the CKD group exhibited a significant increase in glomerular tuft area when compared to control kidneys (Figure 3C). Differences in the mean glomerular tuft area were statistically significant across all kidneys (p<0.001), with an increased mean glomerular tuft area of 8,712±671 µm^2^, as compared to 3,173±100 µm2 in control kidneys. This suggested indirect signs of mesangial expansion secondary to glomerular injury.

### Chronic Kidney Disease mice exhibit severe wound healing impairments

**Figure 4:**
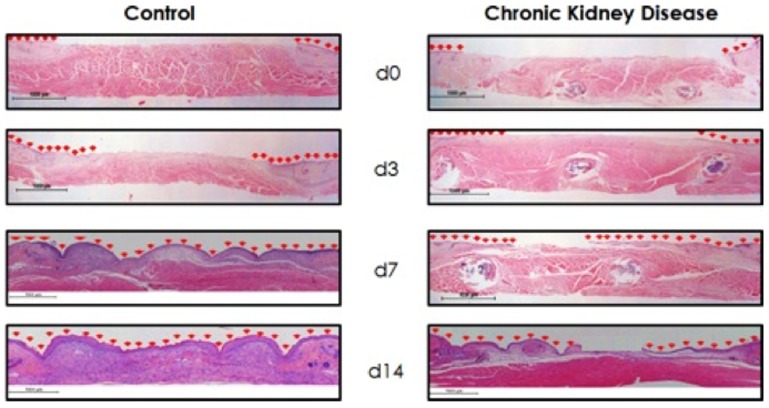
Hematoxylin and eosin (H&E) sections of representative wounds. Wounds were obtained at different harvesting time points, processed, and stained with H&E. Red arrows indicate epithelial keratinocyte migration across the wounds. (Magnification ×20).

**Figure 5:**
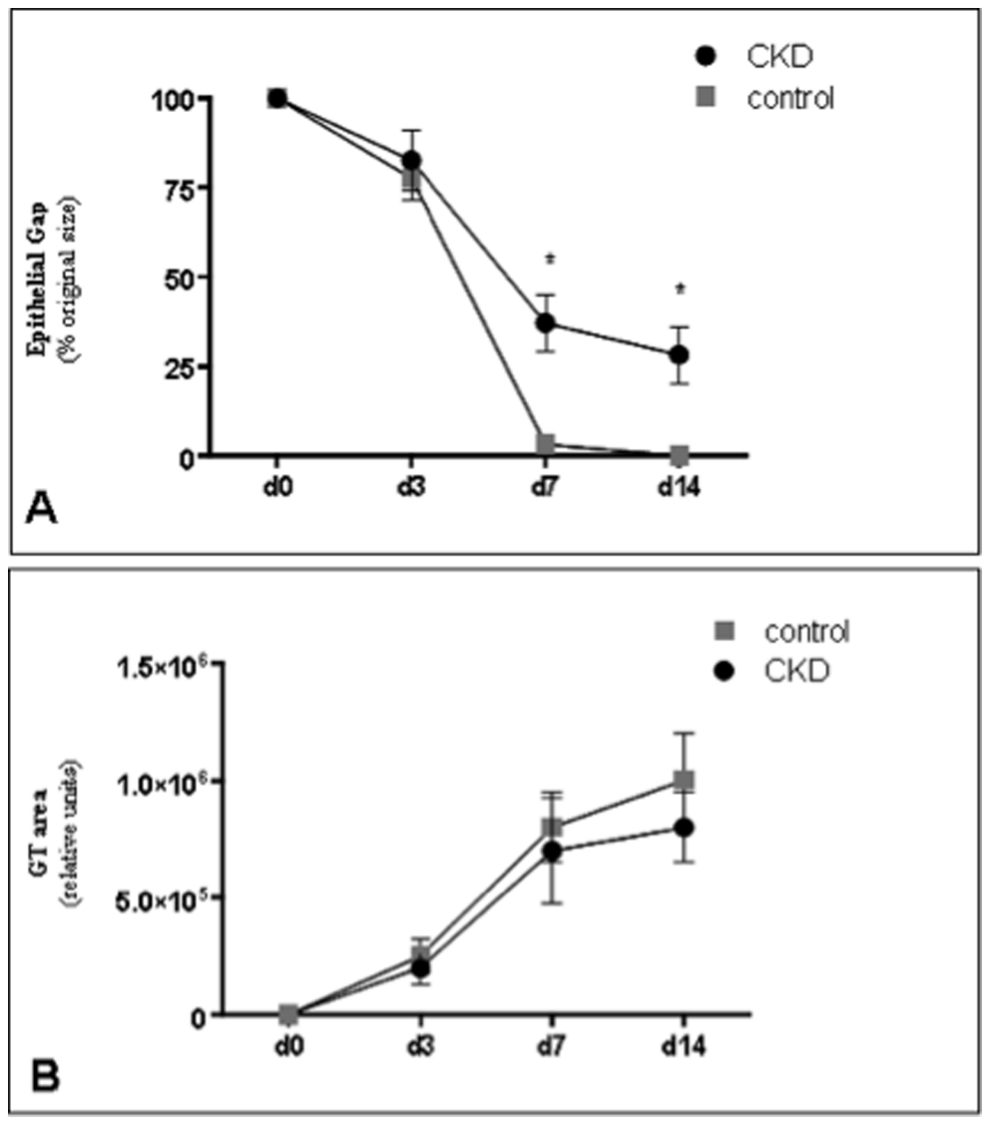
Epithelial gap closure and granulation tissue deposition measurements. **A**) Epithelial gap closure was significantly delayed in the chronic kidney disease model at days 7 and 14. **B**) Granulation tissue deposition exhibited a trend towards being decreased in the chronic kidney disease group. (* *p*<0.05) (n = 6–8 mice/group).

Cross-sectional analysis (Figure 4) of histological wound sections allowed for quantification of the maximal distance between the advancing edges of keratinocyte migration across the wound (epithelial gap), and the digital calculation of all the regions of granulation tissue (granulation tissue area) present in wounds at time of harvest. Control wounds displayed normal reepithelialization kinetics consistent with previously described studies [25], [36]. However, CKD wounds demonstrated statistically significant disruption of normal reepithelialization kinetics and granulation tissue deposition rates. As shown in Figure 5A, the mean percentage of epithelial gap relative to their original size was significantly larger in CKD wounds (day 7 epithelial gap: 37.8±7.0% vs. 3.1±1.9%, *p*<0.05; day 14: 27.3±9.2% vs 0%, *p*<0.05). In addition, although not statistically significant, these CKD affected wounds also exhibited decreased granulation tissue formation (Figure 5B). Animals did not demonstrate any local signs of wound infection or disruption, suggesting that the effects of CKD in these wounds were systemically based. This was further supported by the finding that contralateral wounds in the same animal were found to heal at the same delayed rate. No post-wound splinting fatalities were encountered in any animal.

### CKD wounds exhibit significantly less cellular proliferation and angiogenesis, with concurrent derangements in inflammation, during the early stages of wound healing

**Figure 6:**
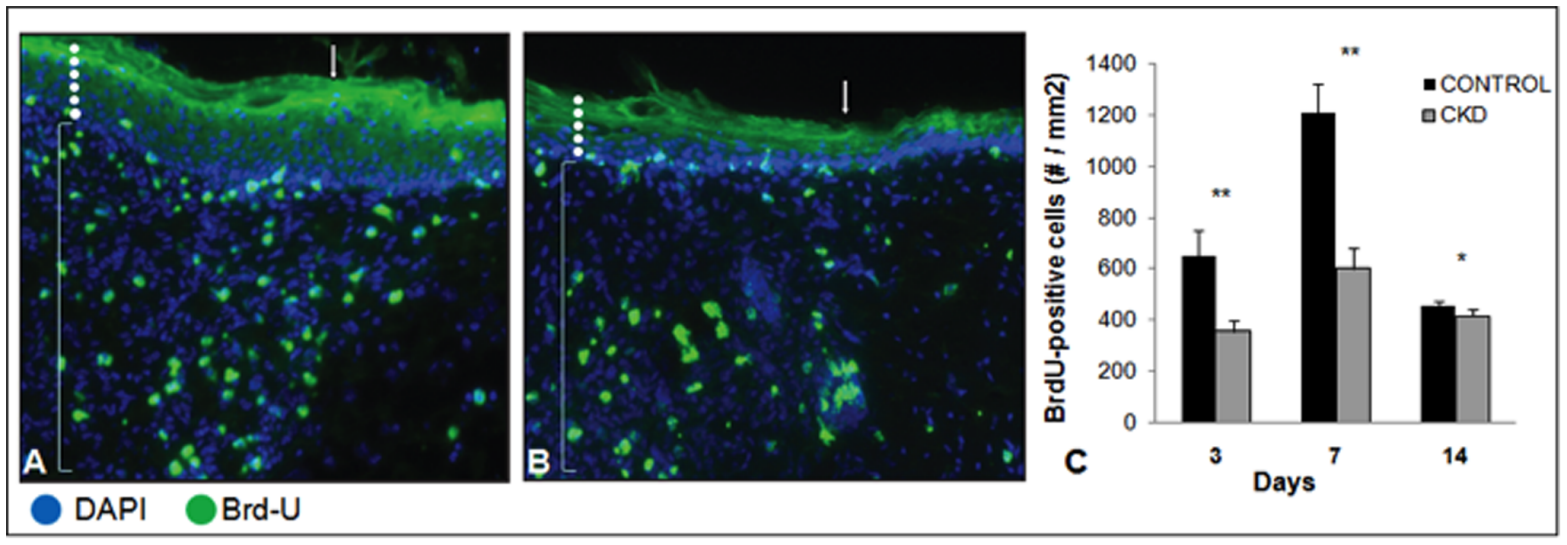
Chronic kidney disease demonstrated significantly less cellular proliferation in the early stages of wound healing. Representative immunofluorescent sections of wound edges stained with BrdU and DAPI in **A**) normal mice and **B**) chronic kidney disease mice at day 7. **C**) Utilizing Tissue Gnostics quantification technology, chronic kidney disease wounds demonstrated significantly less cellular proliferation in the early stages of wound healing. (Arrows indicate wound edges, white dots indicate epidermis, and brackets indicate dermis) (* *p*<0.05, ** *p*<0.01) (Magnification ×200) (n = 6–8 mice/group).

**Figure 7:**
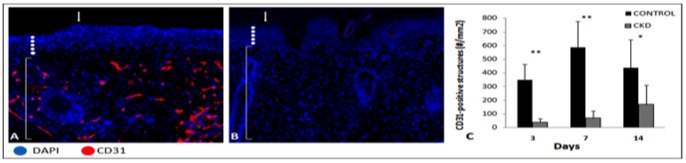
Chronic kidney disease demonstrated a significant reduction in angiogenesis in wound healing tissue. Representative immunofluorescent sections of wound edges stained with CD31 and DAPI in **A**) normal mice and **B**) chronic kidney disease mice at day 7. **C**) Utilizing Tissue Gnostics quantification technology, chronic kidney disease wounds demonstrated significantly less angiogenesis in the early stages of wound healing. (Arrows indicate wound edges, white dots indicate epidermis, and brackets indicate dermis) (* *p*<0.05, ** *p*<0.01) (Magnification ×200) (n = 6–8 mice/group).

Cellular proliferation, angiogenesis and inflammation were assessed at days 3, 7 and 14 by measuring immunofluorescent BrdU, CD31 and CD45-positive cells, respectively, within the entire sampled wounded tissues. Representative images for control (Figure 6A) and CKD (Figure 6B) wounds at day 7 illustrate the differences in BrdU-positive cells within the full thickness wound tissue near the wound edge. Wounds in CKD mice showed significantly less proliferating cells as compared to control wounds in mice with normal renal function (day 3: 355±40 vs. 652±100, *p*<0.001; day 7: 599±81 vs. 1205±117, *p*<0.001; day 14: 414±30 vs. 456±15, *p*<0.05) (Figure 6C). CKD mice also demonstrated a significant reduction in angiogenesis in wound healing tissue when compared to control mice (day 3: 40.5±26.1 vs. 3498.6±114.5, *p*<0.001; day 7: 69.75±53.6 vs. 586.7±191.4, *p*<0.001; day 14: 169.2±140.1 vs. 438.6±203.2, *p*<0.05 (Figure 7C). A digital representation of the sizeable difference in the presence of CD31-positive cells is shown in Figures 7A and 7B for the control and CKD mice at day 7, respectively. Intravascular injection of 8 mice with a fluorescein labeled tetrameric glycoprotein lectin II (FL 1211, Vector Lab, Burlingame, CA) allowed for the visualization and assessment of the presence of vascular endothelium between groups. These results were also consistent with those found following CD31 staining (data not shown).

**Figure 8:**
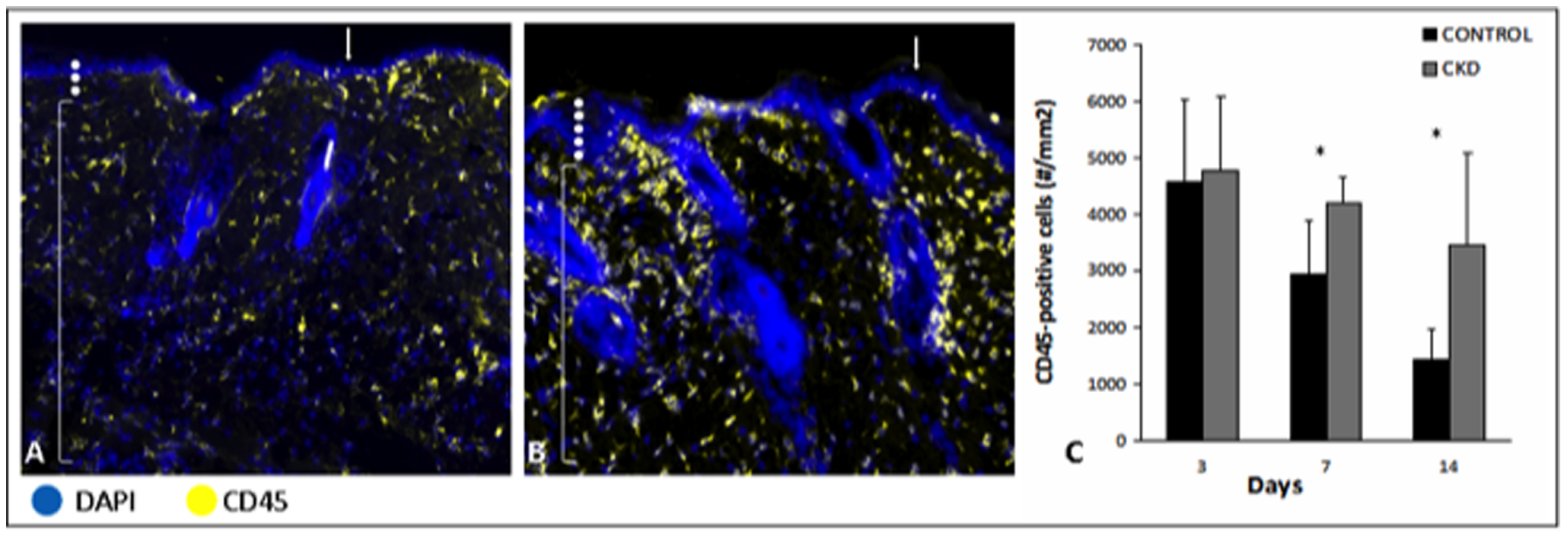
Chronic kidney disease demonstrated significantly increased and maintained inflammation in the early stages of wound healing. Representative immunofluorescent sections of wound edges stained with CD45 and DAPI in **A**) normal mice and **B**) chronic kidney disease mice at day 7. **C**) Utilizing Tissue Gnostics quantification technology, chronic kidney disease wounds demonstrated a significantly increased and maintained inflammation in the early stages of wound healing. (Arrows indicate wound edges, white dots indicate epidermis, and brackets indicate dermis) (* *p*<0.05, ** *p*<0.01) (Magnification ×200) (n = 6–8 mice/group).

**Figure 9:**
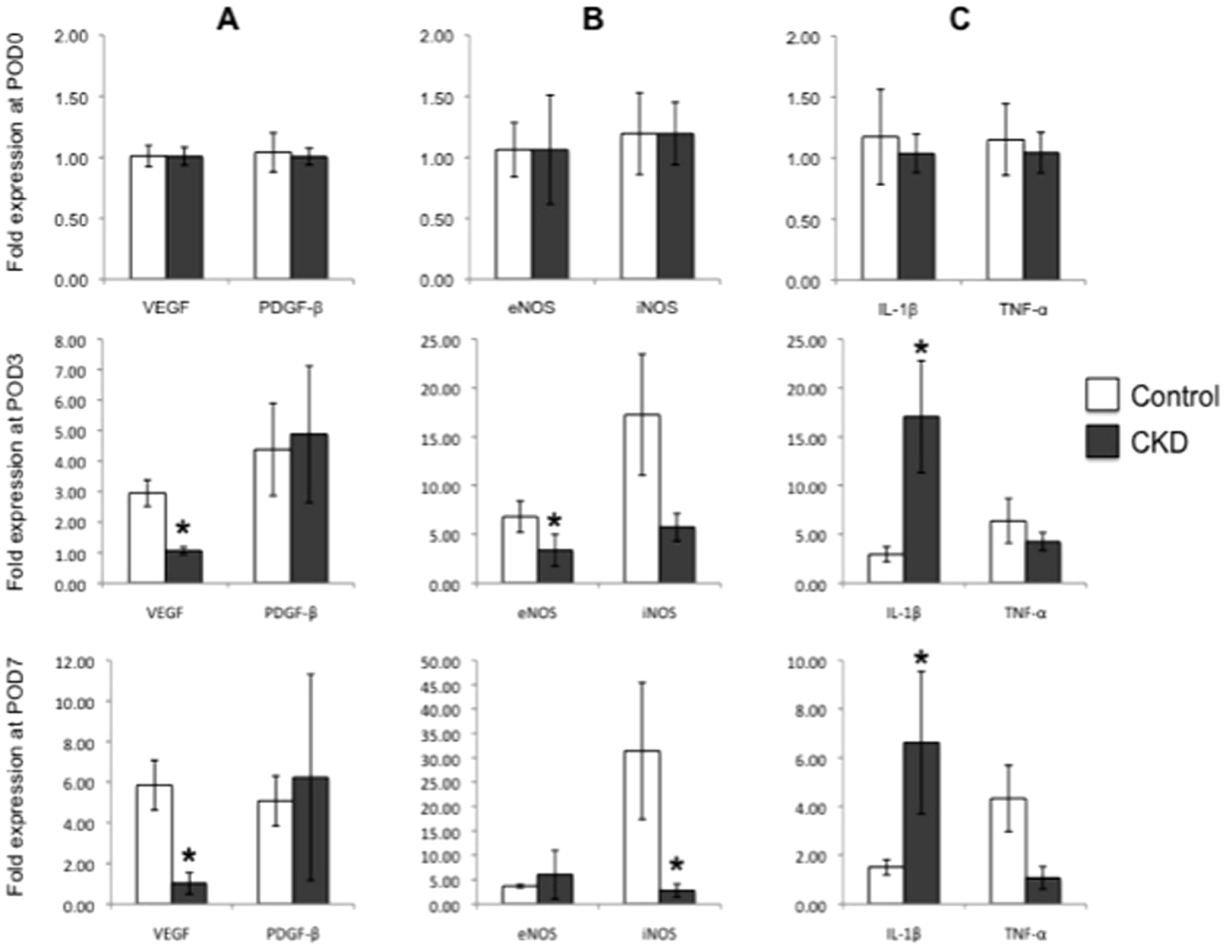
Quantitative reverse-transcription PCR (qRT-PCR) of growth factor, nitric oxide, and inflammatory genese. Genetic analysis of wounds by qRT-PCR exhibited differences in expression of important wound healing related genes due chronic kidney disease. At post-operative day (POD) 3, CKD wounds demonstrate significantly reduced expression of VEGF and eNOS, with an increase in IL-1β. These trends were maintained at POD7, except for a significant decrease in iNOS instead of eNOS. Rows show POD when the tissue was collected, POD0, 3, and 7 at top, middle and bottom, respectively. Genes analyzed are represented by columns: (**A**) growth factor genes, (**B**) nitric oxide genes and (**C**) cytokine genes. (* *p*<0.05) (n = 4 wounds/group).

Interestingly, CKD mice exhibited an increased and maintained inflammatory state during the early stages of wound healing. Digital acquisition of representative control and CKD splinted wounded samples obtained at day 7 are shown in Figures 8A and 8B. CKD wounds revealed a trend towards increased CD45-positive cells at day 3 (4785±1293 vs 4578±1450) (Figure 8C), which by day 7 represented a significant increase in inflammation (4212±456 vs 2940±96, *p*<0.05). This difference was maintained throughout the experiment to day 14 (3461±1617 vs 1434±541, *p*<0.05). Corresponding with our findings on immunofluorescent analysis, qRT-PCR was performed in an effort to evaluate the genetic expression profiles of wounds in CKD mice (Figure 9). Evaluation of critical growth factors and markers of angiogenesis (VEGF, PDGF-β), nitric oxide production (eNOS, iNOS) and systemic inflammation (IL-1β, TNF-α) demonstrated no significant difference between control and CKD mice at baseline on POD0. However on POD3, CKD animals showed a significant reduction in VEGF and eNOS production relative to control (*p*<0.05), with a concurrent increase in the inflammatory marker IL-1β (*p*<0.05). This difference in VEGF and IL-1β production was maintained to day 7 (*p*<0.05), with a progressive decrease in iNOS (*p*<0.05), rather than eNOS, production. In total, these findings, to our knowledge, represent the first evaluation of cellular proliferation, angiogenesis and inflammation in the early stages of wound healing in mice with chronic kidney disease.

## Discussion

Much remains unknown about exactly how skin wounds heal at the molecular level, and furthermore, how they are affected by different pathologies. Patients who have CKD have been long known to have particularly impaired wound healing [17], [27]–[31]. However, the underlying link between the systemic and/or local effects of chronic kidney disease and the observed delay in wound repair requires further elucidation. In the present study, we have modified a murine chronic kidney disease model to better study the effects of this entity on cutaneous wound healing. By combining this established model with our validated splinted excisional wound healing model, we have developed a new model that offers an excellent vehicle for the study of impaired healing and the testing of novel therapeutic interventions. During our initial investigation, we have also confirmed the significant impact of CKD on wound healing, while also identifying associated derangements in angiogenesis, cellular proliferation, and inflammation.

Although previous studies have looked at the association between CKD and delayed wound healing, there are no established models from which continued research has occurred. Nayman [17] first reported in 1966 that uremia from renal failure induced early breakdown of laparotomy wounds in dogs, which was prevented by early and frequent hemodialyses [17]. Meanwhile, Kursh et al [28] demonstrated that uremia had a significant effect on wound healing by measuring wound tensile strength and collagen formation in subcutaneously implanted polyvinyl sponges. However, given the strong correlation between their findings and final body weights, they concluded nutritional factors might be the primary link between uremia and wound healing impairment. Colin et al [29] reported similar results for both abdominal wounds and intestinal anastomoses, although no measurements of the extent of wound healing were performed. Meanwhile, work Shindo et al [30] and Yue et al [31] in uremic rats indicated that the control of surgical infections and diabetes, respectively, might be the most important factors to consider for appropriate wound healing in CKD patients. Given that the most recent of these studies was performed 15 years ago, we believe that the validation of an updated model, which allows for the incorporation of different comorbidities and the study of several different endpoints, is important. Although we have attempted to investigate the mechanistic link between CKD and wound healing, we intend to continue to utilize this model in an attempt to address the growing need for *in vivo* CKD research.

The model we have presented possesses several advantages for the study of CKD-impaired wound healing *in vivo*. In particular, the model's technical ease, along with a lack of local complications, makes it ideal for even the relatively inexperienced investigator to use. The model requires only one surgery to induce CKD, shortening the total experimental time, and more importantly the burden to the animal. Unlike the conventional intraperitoneal anesthetic used for in Gagnon's original model [22], the introduction of inhaled anesthesia allows the mice to better respond to the combination of left nephrectomy and right kidney electro-coagulation in a single procedure, including an almost null mortality rate and a much faster recovery (5–10 minutes v. 30–60 minutes) time. In addition to this, this CKD-inducing surgical model can be tested in any mouse strain, obviating the use of expensive breeds and allowing for a wider horizon of experimentation. However, it is important to note that the degree of uremia that develops in this model is directly proportional to the area of electrocoagulated kidney cortex, which can be subject to technical variability. We attempted to minimize differences in technical performance for this study by having only one of the authors perform all of the surgical procedures.

The incorporation of a reliable, and inexpensive, wound healing model represents another unique advantage of the model that we have presented. In particular, previous work has shown the reproducibility of our excisional wound model with 90% splint maintenance in C57bl/6J mice [25]. This low failure rate was explained by the model utilizing a thin protective dressing around the surgical site, preventing the mice from disrupting the silicone ring splint with their normal grooming behaviors. Consequently, the splints are maintained such that wound contraction is minimized, allowing for an appropriate analysis of epithelial and granulation tissue in-growth. The wrap was also fit to the mouse such that it was ergonomic and did not otherwise affect its normal behavior. With a robust method for evaluating wound healing, intentional modifications to the model can be evaluated and compared in a consistent manner, emphasizing its flexibility for *in vivo* experimentation. Furthermore, the histopathological increase in glomerular tuft area, moderately increased BUN levels, growth retardation, and anemia present in our model are comparable to changes that are seen clinically with CKD, underscoring the model's translatability. The resultant delay in normal wound healing, evidenced by an increased epithelial gap and a decrease in granulation tissue, appropriately models the detrimental, systemic effects of CKD on human wounds, making it a useful model for future wound healing studies.

The consistency of our model was further established by our immunofluorescence and qRT-PCR findings. With a delay in wound healing, an associated decrease in cellular proliferation (BrdU) and angiogenesis (CD31) was to be expected. A concurrent significant decrease in VEGF on qRT-PCR further emphasized the detrimental impact of CKD on angiogenesis. Meanwhile, the distinct change in host inflammatory response was a surprising finding. Highlighted by a significant increase in CD45-positive cells and IL-1β that was maintained over time, we believe that these findings are indicative of the potential importance of inflammatory mediators in the systemic effects of CKD on wound healing. Interestingly, the expression of systemic markes IL-1β and TNF-α did not correlate, despite both being markers of systemic inflammation. Rather than being an unclear discrepancy, this may represent a specific inflammatory pathyway that is associated with CKD, warranting further investigation. Furthermore, IL-1β is expressed by a larger variety of cells beyond macrophages, including fibroblasts, and has been seen to have a different expression pattern than TNF-α in other wound-related settings, such as with bacterial biofilm [37]. Taken individually, perturbations in angiogenesis and inflammation can have significant systemic effects given the ubiquity of these processes within the human body. In particular, their importance to normal wound healing cannot be emphasized enough. Therefore, with CKD having a demonstrable impact on both of these systems, our findings validate its importance to the pathophysiology of non-healing wounds. Further studies aimed at elucidating the molecular pathways responsible for these changes may identify potential avenues for therapeutic intervention, which can then be quickly translated into the clinical arena.

Despite a rigorous, scientific approach, we acknowledge the limitations of our study. In particular, although we were consistently able to induce a moderate CKD phenotype, we did not evaluate its individual impact on our different endpoints, including wound healing. Given the spectrum of severity seen clinically, the ‘dose-dependent’ impact decreasing renal function in CKD would be of interest for future studies. Also, given our goal of establishing and validating an initial model, we did not extend our experiments to other strains of mice. As previously mentioned, the inherent flexibility of our model provides a platform for studying CKD-impaired wound healing against different genetic backgrounds. For example, utilizing a diabetic mouse strain would allow for further characterization of the complex interplay between CKD, diabetes and wounds, the three of which are commonly found within a single patient. Meanwhile, the introduction of bacteria into our wounds, specifically biofilm, would help to address another essential pillar of chronic wound pathogenesis. Given this potential for extensive experimentation, we chose to limit our study to the findings that have been presented. It is our hope that the information presented has provided greater insight into the relationship between CKD and wound healing, while establishing a foundation from which investigators can continue answer questions related to wound and CKD pathophysiology.
